# Clonal Dissemination of *Aeromonas hydrophila* With Binary Carriage of *bla*_KPC-2_-Bearing Plasmids in a Chinese Hospital

**DOI:** 10.3389/fmicb.2022.918561

**Published:** 2022-07-08

**Authors:** Zhijiang Xu, Weiyi Shen, Rong Zhang, Jiachang Cai

**Affiliations:** Clinical Microbiology Laboratory, The Second Affiliated Hospital of Zhejiang University School of Medicine, Zhejiang University, Hangzhou, China

**Keywords:** *Aeromonas hydrophila*, *bla*_*KPC*−2_, clonal spread, clinical setting, genetic environment

## Abstract

Dissemination of the *Klebsiella pneumoniae* carbapenemase (KPC)-encoding gene among Enterobacterales is common but relatively rare in *Aeromonas* spp. In this study, we characterized two KPC-2-producing *Aeromonas hydrophila* strains (Ah2101 and Ah2111), each isolated from a patient in different intensive care units (ICUs) of a Chinese hospital. Whole-genome sequencing (WGS) revealed simultaneous carriage of the *bla*_KPC−2_ and *imiH* genes, both of which encode high-level carbapenem resistance in these two *A. hydrophila* isolates. The two isolates were shown to be clonally related and each isolate harbored two distinguishable *bla*_KPC−2_-bearing plasmids, only one of which was transferrable to *A. hydrophila*, but not *Escherichia coli* EC600 *via* conjugation. The genetic element that contains *bla*_KPC−2_ in these two plasmids, namely IS*Kpn27*-Δ*bla*_TEM−1_-*bla*_KPC−2_-IS*Kpn6*, was structurally identical, commonly detected in Enterobacterales, and associated with Tn*3*-based transposons. In addition, more than sixty putative genes that encode various virulence factors were identified in these two *A. hydrophila* isolates. This is the first study that reports clonal dissemination of carbapenem-resistant *A. hydrophila* strains carrying structurally different *bla*_KPC−2_-bearing plasmids. Further investigation is warranted to monitor the future transmission of *bla*_KPC−2_-bearing plasmids in *A. hydrophila* in clinical settings.

## Introduction

Global dissemination of carbapenemase-producing organisms (CPOs) is an emerging public health issue, with the potential of further compromising the effectiveness of treatment of bacterial infection. *Klebsiella pneumoniae* carbapenemase (KPC), which is encoded by the plasmid-borne gene *bla*_KPC_, possesses a large and shallow active site, allowing such an enzyme to accommodate and hydrolyze a wide range of β-lactam molecules, including cephalosporins, monobactams, and carbapenems (Bush and Bradford, 2020). KPC producers are not only resistant to most β-lactams but also display a multidrug resistance phenotype as they also carry additional resistance genes which confer resistance to many of the currently available first-line therapeutic alternatives (Munoz-Price et al., 2013). Meanwhile, infections caused by KPC-producing organisms are associated with high mortality rates of up to 51%, thus imposing a heavy burden on the healthcare sector (Giacobbe et al., 2015). Since its first description in 1996 (Yigit et al., 2001), genetic elements encoding these types of carbapenemase have spread extensively among Gram-negative bacteria. In China, such an enzyme was identified for the first time in a *K. pneumoniae* isolate collected in Zhejiang Province in 2004 (Wei et al., 2007). KPC-2 is the most prevalent carbapenemase in China, with *K. pneumoniae* being the most common host species that produces such an enzyme. The high mobility of genetic elements carrying the *bla*_KPC_ gene implies that such elements can be readily disseminated to other Gram-negative bacteria including those of the genus *Aeromonas* (Picao et al., 2013).

*Aeromonas* spp. are ubiquitous organisms widely distributed in various aquatic environmental niches and may act as opportunistic pathogens that cause a number of self-limiting gastrointestinal infections and extra-intestinal infections, including those of the skin, soft tissue, and wound, as well as pneumonia and bacteremia (Janda and Abbott, 2010; Nolla-Salas et al., 2017). In recent years, multidrug-resistant *Aeromonas* strains have been isolated from clinical, animal, food, and environmental samples (Tang et al., 2020). In particular, carbapenem-resistant strains exhibit a prevalence rate ranging from 0.5 to 7.7% in different countries. *Aeromonas* spp. producing KPC-2 carbapenemases have also been successively isolated in the past decades from environmental samples in Brazil (Montezzi et al., 2015), the United States (van Duin and Doi, 2017; Mathys et al., 2019), Japan (Sekizuka et al., 2019), and China (Xu et al., 2018; Hu et al., 2019), indicating that the strains of this genus may serve as a vector for dissemination of the *bla*_KPC−2_ gene. Although KPC-2-producing *Aeromonas* spp. were once isolated from routine perirectal surveillance in hospitalized adult patients in the United States (Hughes et al., 2016), this type of strain was rarely detected in clinical settings. Here, we report the recovery of two structurally different *bla*_KPC−2_-bearing plasmids from two clinical isolates of *A. hydrophila*. Genome analysis based on whole-genome sequencing (WGS) data was performed to reveal the genetic relatedness between these two strains and shed light on the genetic environment of *bla*_KPC−2_ genes, as well as the molecular features of plasmids that harbor such genes.

## Materials and Methods

### The Patients and Bacterial Strains

Two *Aeromonas* spp. (strains Ah2101 and Ah2111) were isolated from two patients hospitalized in intensive care units (ICUs) of The Second Affiliated Hospital of Zhejiang University School of Medicine in 2021. The species identity of the test strains was determined by MALDI-TOF MS (Bruker Daltonik GmbH, Bremen, Germany). A comparison of genomes between the reference strain (*A. hydrophila* OnP3.1, GenBank accession no. NZ_CP050851.1) and the two isolates was performed by using the average nucleotide identity (ANI) calculator (http://enve-omics.ce.gatech.edu/ani/index). The result showed that the ANI values between *A. hydrophila* OnP3.1 and strain Ah2101, and between *A. hydrophila* OnP3.1 and strain Ah2111 were 96.86% and 96.87%, respectively, confirming that both isolates belonged to *A. hydrophila*. This study was approved by the Ethics Committee of The Second Affiliated Hospital of Zhejiang University School of Medicine, and consents were given by the patients or their families.

Patient A, a 52-year-old male, was admitted to the emergency ICU due to skull fractures and traumatic intracranial hemorrhage caused by a traffic accident in January 2021. After seven days of empirical therapy with piperacillin/tazobactam (8:1, 4.5 g IV for every 8 h), the patient developed complications that involved severe intracranial infection and the treatment strategy was then changed to the use of meropenem (1 g IV for every 8 h). On the second day of administration, the cerebrospinal fluid culture grew two carbapenem-resistant strains of *A. hydrophila* (strain Ah2101) and *Acinetobacter baumannii*. A course of tigecycline treatment (100 mg IV for every 12 h) was then applied according to the antimicrobial susceptibility results, and these two isolates were cleared from the cerebrospinal fluid after 4 days of treatment. Unfortunately, the patient became critically ill without indications of the benefit of another operation upon thorough assessment; only supportive treatment was given and the prognosis was grave. Finally, the patient was discharged at the request of the family.

Patient B, a 75-year-old female with mitral stenosis and cardiac dysfunction, underwent mitral valve replacement and was admitted to the cardiac ICU in September 2021. After hospitalization for 1 month, the patient suffered from abdominal pain and developed a fever. CT examination suggested cholecystitis and peritoneal effusion. Cholecystostomy and abdominal catheterization/drainage were then performed and a course of imipenem (500 mg IV for every 8 h) was started. On the 25th day of treatment, a carbapenem-resistant *A. hydrophila* strain, Ah2111, was isolated from the drainage sample. Fortunately, the patient's condition gradually improved upon receipt of various supportive treatments and continuous drainage of peritoneal effusion. The second culture of the drainage fluid for 8 days after the initial isolation was negative for *A. hydrophila*. The drainage volume decreased gradually and the drainage tube was removed 2 weeks later. The patient was ultimately discharged from the hospital.

### Antimicrobial Susceptibility Testing (AST)

The minimal inhibitory concentrations (MICs) of 15 antimicrobial agents, including imipenem, meropenem, ertapenem, ceftazidime, cefotaxime, cefepime, piperacillin/tazobactam, cefoperazone/sulbactam, aztreonam, ciprofloxacin, amikacin, chloramphenicol, tetracycline, trimethoprim/sulfamethoxazole, and ceftazidime/avibactam were determined using the broth microdilution method and interpreted according to the Clinical and Laboratory Standards Institute (CLSI) guidelines (Clinical Laboratory Standards Institute, 2015, 2021). *Escherichia coli* ATCC 25922, *E. coli* ATCC 35218, and *Pseudomonas aeruginosa* ATCC 27583 were used as the quality control strains in parallel.

### Whole-Genome Sequencing (WGS) and Genome Analysis

The genomic DNA extracted from *A. hydrophila* Ah2101 and Ah2111 was subjected to WGS by using the Illumina NovaSeq 6000 platform with a paired-end read length of 150 bp (NEBNext Ultra DNA Library Prep Kit) and the sequencing data were *de novo* assembled into contigs using SPAdes version 3.13.1 (Bankevich et al., 2012). *A. hydrophila* strain Ah2111 was also subjected to sequencing by the Oxford Nanopore PromethION 48 system; hybrid genome assembly of both short and long reads was carried out by using the Unicycler version 0.4.4 software (Wick et al., 2017). Assembled genomes and *bla*_KPC−2_-bearing plasmids were annotated by the RAST server (https://rast.nmpdr.org/) (Overbeek et al., 2014). A pairwise comparison of genomes and variant callings for single-nucleotide polymorphisms (SNPs) was conducted using Snippy version 4.4.5 with default settings (https://github.com/tseemann/snippy). Carriage of the antimicrobial resistance genes and the Inc-type of plasmids for the assembly scaffolds were determined with default settings by ResFinder 3.2 (Zankari et al., 2012) and PlasmidFinder 2.0 (Carattoli et al., 2014), respectively, at the Center for Genomic Epidemiology (https://cge.cbs.dtu.dk/services/). Visualization of genome comparison for *bla*_KPC−2_-harboring plasmids was implemented using BRIG (version 0.95) (Alikhan et al., 2011). The virulence factors in *A. hydrophila* were identified using the VFanalyzer (http://www.mgc.ac.cn/VFs/main.htm).

### Transferability of the *Bla*_KPC-2_ Gene

Conjugation experiments based on the filter mating method were performed to access the transferability of the plasmid-mediated *bla*_KPC−2_ gene; a *bla*_KPC−2_-positive *K. pneumoniae* strain K1 reported in our previous study was used as the positive control strain (Cai et al., 2008). Apart from rifampicin-resistant *E. coli* EC600, an induced-rifampicin-resistant *A. hydrophila* (strain AhRF100), which was susceptible to carbapenems (Table 1), was also employed as the recipient strain. The putative transconjugants that grew on the MacConkey agar plates supplemented with 50 mg/L rifampicin and 0.3 mg/L meropenem were identified by MALDI-TOF MS and screened for the acquisition of the *bla*_KPC−2_ gene by PCR. The conjugation frequency was calculated as the ratio of the number of transconjugants to the number of donors.

**Table 1:** Antimicrobial susceptibility of *A. hydrophila* Ah2101 and Ah2111, the corresponding transconjugants, and plasmid-eliminated strains.

**Strain^a^**	**MICs (mg/L)^b^**														
	**IPM**	**MEM**	**ETP**	**CAZ**	**CTX**	**FEP**	**TZP**	**SCF**	**ATM**	**AK**	**CIP**	**C**	**TE**	**SXT**	**CZA**
Ah2101	>128	>128	>128	>128	>128	>128	>256/4	256/128	>128	>128	32	2	4	≤1/19	≤0.5/4
Ah2111	>128	>128	>128	>128	>128	>128	>256/4	256/128	>128	>128	16	2	4	≤1/19	≤0.5/4
Ah2101-PE	16	16	128	>128	>128	64	≤8/4	≤8/4	128	≤4	32	2	4	≤1/19	≤0.5/4
Ah2111-PE	16	32	128	>128	>128	64	≤8/4	≤8/4	128	≤4	32	2	4	≤1/19	≤0.5/4
AhRF100	≤0.5	≤0.5	≤0.5	≤1	≤1	≤1	≤8/4	≤8/4	≤1	≤4	≤0.25	≤1	≤1	≤1/19	≤0.5/4
AhRF100-2101-TC	16	64	128	32	>128	128	256/4	128/64	128	>128	≤0.25	≤1	≤1	≤1/19	≤0.5/4
AhRF100-2111-TC	16	64	128	32	>128	64	128/4	128/64	128	>128	≤0.25	≤1	≤1	≤1/19	≤0.5/4

a *PE, A. hydrophila strains whose bla_KPC−2_-bearing plasmids were eliminated; TC, A. hydrophila transconjugants carrying the bla_KPC−2_-bearing plasmid*.

b *IPM, imipenem; MEM, meropenem; ETP, ertapenem; CAZ, ceftazidime; CTX, cefotaxime; FEP, cefepime; TZP, piperacillin/tazobactam; SCF, cefoperazone/sulbactam; ATM, aztreonam; CIP, ciprofloxacin; AK, amikacin; C, chloramphenicol; TE, tetracycline; SXT, trimethoprim/sulfamethoxazole; CZA, ceftazidime/avibactam. For piperacillin/tazobactam and ceftazidime/avibactam, the tazobactam and avibactam were tested at a fixed concentration of 4 mg/L. For cefoperazone/sulbactam, the combination was tested with concentrations of 2:1 ratio (antibiotic: inhibitor)*.

### Plasmid Elimination

Plasmid elimination assays were performed for both *A. hydrophila* Ah2101 and Ah2111 with repeated sodium dodecyl sulfate (SDS) treatment in Luria-Bertani (LB) broth. For every passage, 100 μl of the culture was added to a subculture of 5 ml fresh LB broth containing 4% SDS, followed by overnight incubation at 37°C with shaking (200 rpm). The culture (20 μl) was then placed and streaked on LB plates, from which 30 single colonies were selected for assessment of carriage of the *bla*_KPC−2_ gene by PCR. The serial transfer and SDS treatment experiment were conducted until the *bla*_KPC−2_-bearing plasmids were eliminated from the parent isolates.

### Nucleotide Sequence Accession Numbers

The genome of *A. hydrophila* Ah2101 and the complete genome sequence of *A. hydrophila* Ah2111 containing one chromosome and seven plasmids have been deposited into the NCBI database under BioProject accession number PRJNA823662 and GenBank accession numbers JALKAG000000000 and CP095280 to CP095287.

## Results

### Antimicrobial Susceptibility Results

*A. hydrophila* strains Ah2101 and Ah2111 exhibited similar antimicrobial susceptibility profiles characterized by high-level resistance to β-lactams including carbapenems. MIC values of imipenem, meropenem, ertapenem, ceftazidime, cefotaxime, cefepime, piperacillin/tazobactam, cefoperazone/sulbactam, and aztreonam were above 128 mg/L. Both *Aeromonas* strains were also resistant to ciprofloxacin and amikacin but remained susceptible to ceftazidime/avibactam, chloramphenicol, tetracycline, and trimethoprim/sulfamethoxazole (Table 1).

### Whole-Genome and Phylogenetic Analysis

The genome of the two *A. hydrophila* strains, both of a size of ~5.2 Mbp, was obtained. Whole-genome analysis revealed carriage of a variety of β-lactamase-encoding genes in both strains, including *bla*_KPC−2_, Δ*bla*_TEM−1_, *bla*_PER−3_, *ampH*, and *imiH*. Among these genes, *imiH* is known to encode a species-specific metallo-β-lactamase (MBL), which is a member of the CphA family that selectively hydrolyzes penems and carbapenems rather than penicillin and cephalosporins (Bush and Bradford, 2020). Multiple antimicrobial resistance determinants were also detected, conferring resistance to quinolone (*qnrS2*), chloramphenicol (*catB3*), macrolide [*mph*(A)], quaternary ammonium compounds (*qacE*), sulfonamides (*sul1*), and aminoglycosides [*armA, aadA16, aac(6*′*)-Ib*, and *rmtB*]. These two isolates, which share similar virulence gene profiles, produce various virulence factors, including pili, polar flagella, the type VI secretion system (T6SS), Exe T2SS, aerolysin, hemolysin, the repeat in toxin A (RtxA), and heat-stable cytotonic enterotoxin, which are responsible for adherence, effector delivery, motility, and production of exotoxin during the infection process. In addition, several genes encoding nutritional or metabolic factors which may be associated with bacterial virulence were identified (Supplementary Table S1).

To determine the genetic relatedness of *A. hydrophila* strains Ah2101 and Ah2111, a genome-based phylogenetic analysis was performed. The result showed that the two isolates differed by only eight SNPs and a deletion of a 33-bp fragment among a total of 5,079 putative genes, indicating that they belonged to the same clone despite the fact that they were recovered from two patients in different wards in the same hospital at dates that were 10 months apart. The eight SNPs were detected in one non-coding region and seven coding sequences (CDS), generating missense mutations in genes encoding the histidine permease YuiF, a methyl-accepting chemotaxis sensor/transducer protein, the MSHA biogenesis protein MshG, the sensory histidine kinase CreC, and a tail-specific protease precursor, as well as synonymous mutations in genes that encode a transcriptional regulator YidZ and an uncharacterized protein YfjI. In contrast, the 33-bp deletion was detected in a CDS that encodes a putative lipoprotein (Table 2).

**Table 2:** Comparative genetic analysis of *A. hydrophila* Ah2101 and Ah2111.

**No**.	**Type**	**Region**	**Effect**	**Gene**	**Product**
1	SNP	CDS	Missense	*yuiF*	Histidine permease YuiF
2	SNP	CDS	Missense	Unnamed	Methyl-accepting chemotaxis sensor/transducer protein
3	SNP	CDS	Missense	Unnamed	MSHA biogenesis protein MshG
4	SNP	CDS	Missense	*creC*	Sensory histidine kinase CreC of two-component signal transduction system CreBC
5	SNP	CDS	Missense	*prc*	Tail-specific protease precursor
6	SNP	CDS	Synonymous	*yidZ*	Transcriptional regulator YidZ, LysR family
7	SNP	CDS	Synonymous	*yfjI*	Uncharacterized protein YfjI
8	SNP	Non-coding region	None	none	none
9	Deletion	CDS	Disruptive in-frame deletion	Unnamed	Putative lipoprotein

### Molecular Characteristics and Transferability of *Bla*_KPC-2_-Bearing Plasmids

**Figure 1:**
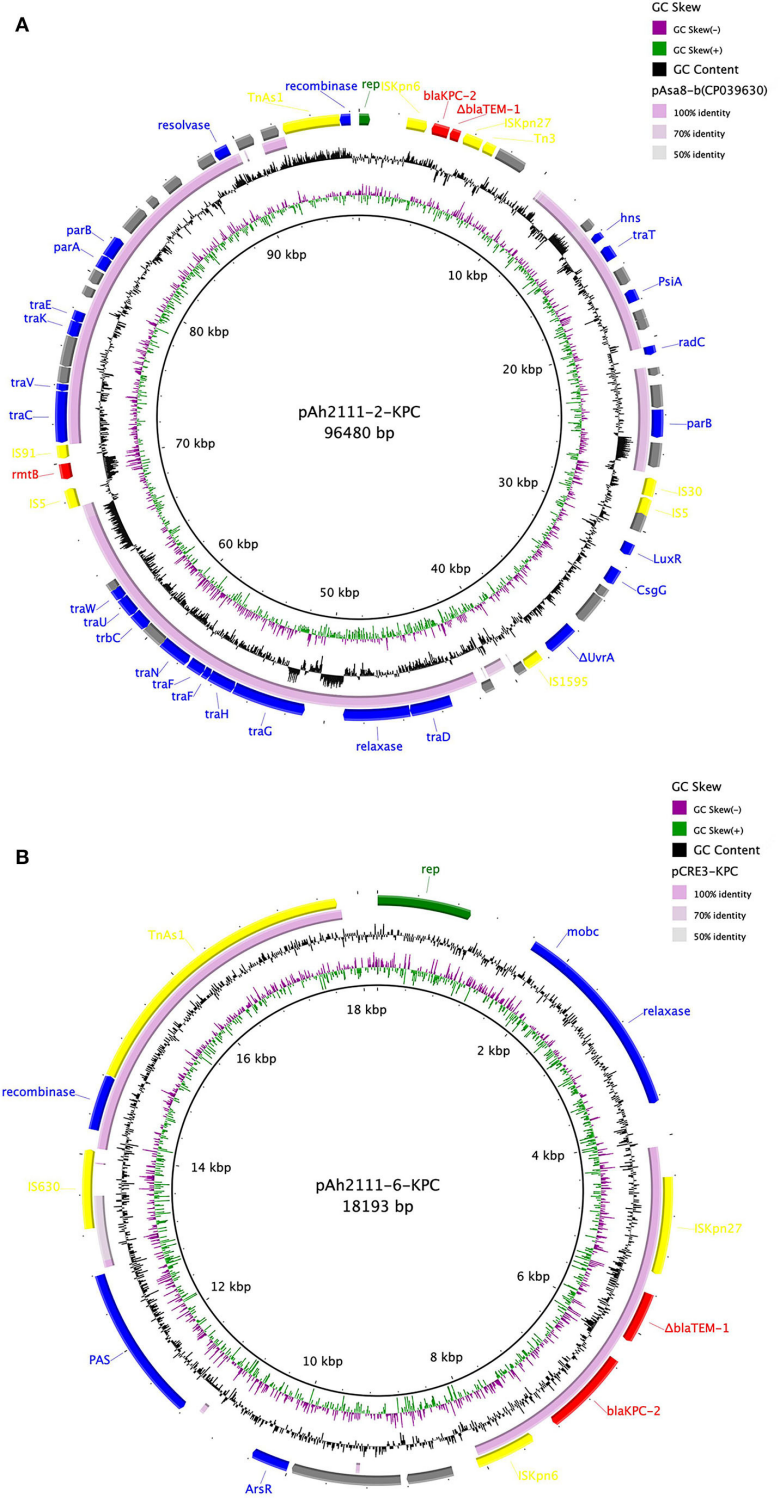
Comparison of two *bla*_KPC−2_-carrying plasmids pAh2111-2-KPC **(A)** and pAh2111-6-KPC **(B)** with the reference plasmids. The five circles from inside to outside displayed the scale in kilobase pairs, the GC skew, the GC content, level similarity to the reference plasmids, and annotation of the plasmids tested in this study, respectively.

To determine the location and genetic context of the *bla*_KPC−2_ gene, *A. hydrophila* Ah2111, which was closely related to strain Ah2101 according to the result of phylogenetic analysis which showed that they exhibited differences of only eight SNPs and one deletion, was subjected to long-read sequencing, and the complete genome of the size of around 5.3 Mbp was obtained. The complete sequence of *A. hydrophila* Ah2111 comprised a chromosome of 5,035,848 bp in length as well as seven plasmids. Two *bla*_KPC−2_-carrying plasmids designated as pAh2111-2-KPC and pAh2111-6-KPC, respectively, were identified and found to differ significantly in size (96,480 vs. 18,193 bp) and plasmid structure. Apart from *bla*_KPC−2_, an *rmtB* gene, which can mediate the expression of high-level resistance to aminoglycosides including amikacin, was also located in the plasmid pAh2111-2-KPC. The *qnrS2* gene was another plasmid-borne antimicrobial resistance gene located in a third plasmid, whereas the other resistance genes were chromosomally located. Based on the sequences of pAh2111-2-KPC and pAh2111-6-KPC, two structurally similar *bla*_KPC−2_-carrying plasmids were obtained from *A. hydrophila* Ah2101 by PCR mapping. Further analysis showed that the homology of the backbone for plasmid pAh2111-2-KPC exhibited the highest degree of sequence homology to the plasmid pAsa8-b (GenBank accession no. CP039630) in the *Aeromonas caviae* strain R25-2, with 97.94% identity (Figure 1A). A cluster of *tra* genes was conserved in plasmids recovered from *Aeromonas* spp., suggesting that this plasmid originated from this genus. Another *bla*_KPC−2_-carrying plasmid, pAh2111-6-KPC, shared 99.54% similarity in 42% of the sequence with plasmid pCRE3-KPC (GenBank accession no. MH919378), which was from *Citrobacter braakii*, including the *bla*_KPC−2_ gene locus in the length of 3,667 bp (Figure 1B). Sequence alignment of the replication gene *rep* of this plasmid revealed 95.24% identity to a variety of plasmid-borne *rep* genes from *Aeromonas* spp. and the lack of the *tra* module, suggesting that this plasmid was non-conjugative. Unlike some IncP-6-type *bla*_KPC−2_-carrying plasmid in *Aeromonas* spp. (Hu et al., 2019; Sekizuka et al., 2019), the two plasmids tested in this study were found to belong to an unknown Inc-type.

### Transferability and Elimination of *Bla*_KPC-2_-Bearing Plasmids

Conjugation experiments were conducted to evaluate the intra- and inter-species transferability of two *bla*_KPC−2_-bearing plasmids. Both *A. hydrophila* Ah2101 and Ah2111 were capable of transferring the *bla*_KPC−2_ gene to *A. hydrophila* AhRF100, with a conjugation efficiency of 2.9 × 10^−4^ and 7.7 × 10^−4^, respectively. The functionality of *bla*_KPC−2_ genes in *A. hydrophila* transconjugants (*A. hydrophila* AhRF100-2101-TC and AhRF100-2111-TC) was further verified by antimicrobial susceptibility tests, with results showing that both transconjugant strains exhibited elevated MICs of carbapenems (ranging from 16 to 128 mg/L) when compared with the recipient strain (Table 1). To distinguish between the two *bla*_KPC−2_-bearing plasmids, PCR amplification using two pairs of primers that target the *rep* genes of plasmids pAh2111-2-KPC and pAh2111-6-KPC, respectively, was performed. Both *A. hydrophila* transconjugants of *A. hydrophila* Ah2101 and Ah2111 were only positive for the *rep* gene of plasmid pAh2111-2-KPC. This finding, which was consistent with the high-level amikacin resistance phenotype observed in transconjugants, confirmed the horizontal transferability of pAh2111-2-KPC between two different *A. hydrophila* strains. However, conjugative transfer of carbapenem resistance to *E. coli* EC600 was unsuccessful.

Both *bla*_KPC−2_-carrying plasmids in *A. hydrophila* Ah2101 and Ah2111 were eliminated, followed by the SDS treatment in three passages. The MICs of imipenem and meropenem of filial strains for *A. hydrophila* Ah2111 and Ah2101 (*A. hydrophila* Ah2101-PE and Ah2111-PE) decreased by more than 8-fold when compared to parent strains. In addition, susceptibility of the two plasmid-eliminated strains to piperacillin/tazobactam, cefoperazone/sulbactam, and amikacin was restored (Table 1).

### Genetic Context of the *Bla*_KPC-2_ Genes

Analysis of the genetic environment surrounding the *bla*_KPC−2_ genes that suggested a highly similar genetic structure of “IS*Kpn27*-Δ*bla*_TEM−1_-*bla*_KPC−2_-IS*Kpn6*” was shared by plasmid pAh2111-2-KPC and pAh2111-6-KPC of 3,984 bp and 3,667 bp, respectively. In plasmid pAh2111-2-KPC, an integration structure of Tn*3*-based transposon was found located upstream of IS*Kpn27* and formed the transposable unit “ΔTn*3*-IS*Kpn27*-Δ*bla*_TEM−1_-*bla*_KPC−2_-IS*Kpn6*,” which was common in *bla*_KPC_-bearing plasmids in Enterobacterales. An example is *K. pneumoniae* strain WCHKP13F2 isolated from Chengdu, China (GenBank accession no. CP028389.3). A previous study demonstrated that Tn*3*-based transposons might mediate the dissemination of the *bla*_KPC−2_ gene between different Enterobacterales in China (Shen et al., 2009). As this Tn*3*-based transposon cannot be conjugated to *E. coli* EC600, it is conceivable that this Enterobacterales-bearing genetic element plays a role in mediating mobilization and further dissemination of the *bla*_KPC−2_ gene to plasmids in *Aeromonas* spp.

## Discussion

*Aeromonas* spp. thrive in aquatic environments and are regarded as enteric pathogens of limited pathogenicity (Janda and Abbott, 2010). In the past few decades, isolation of KPC-producing *Aeromonas* spp. from environmental samples is uncommon; similarly, nosocomial infections due to *Aeromonas* spp. are rare. In this study, we reported the clonal spread of two *bla*_KPC−2_-carrying *A. hydrophila* strains recovered from clinical samples of patients in ICUs with prior carbapenem exposure. The emergence and dissemination of these isolates were possibly associated with antibiotic therapy. Previous reports on *bla*_KPC−2_-carrying *Aeromonas* spp. in clinical settings have only involved sporadic isolation of a single clinical strain (Tang et al., 2020). Our study showed that there is a risk for clonal dissemination of such strains, presumably due to the selective pressure of β-lactams. Furthermore, a 10-month interval between the isolation of *A. hydrophila* strains Ah2101 and Ah2111 indicated that these strains may act as *bla*_KPC−2_ gene reservoirs that persist for a long period of time in the hospital environment, potentially causing life-threaten infectious diseases, especially among ICU patients who have weakened immunity and often require antimicrobial treatment.

Two *bla*_KPC−2_-carrying plasmids of different sizes harbored by *A. hydrophila* Ah2101 and Ah2111 were also characterized and a similar genetic structure of “IS*Kpn27*-Δ*bla*_TEM−1_-*bla*_KPC−2_-IS*Kpn6*” was identified. This structure was frequently detected in Enterobacterales and commonly associated with a Tn*3*-based transposon, which had been reported in a variety of IncP-6 plasmids of *Aeromonas* strains recovered from both environmental and clinical samples (Hu et al., 2019; Sekizuka et al., 2019). Most of these types of plasmids were detected in China; however, both *bla*_KPC−2_-carrying plasmids in this study belonged to an unknown Inc-type. Furthermore, conjugation experiments with *E. coli* EC600 and *A. hydrophila* AhRF100 as the recipient strains yielded the opposite results, suggesting that horizontal transfer of the plasmid pAh2111-2-KPC was restricted to the same species or genus. Given the high degree of sequence homology between the backbone of pAh2111-2-KPC and that of plasmids from *Aeromonas* spp., it is assumed that the spread of the *bla*_KPC−2_ gene to *Aeromonas* spp. might not be mediated by the transmission of the *bla*_KPC−2_-carrying plasmids from Enterobacterales.

The structural plasticity of Tn*3*-based transposons was also demonstrated previously through the identification of various truncated *bla*_TEM−1_ genes which typically constitute the Tn*3* transposons, with or without carriage of Tn*3*-transposase and resolvase-encoding variant genes (Shen et al., 2009; Yang et al., 2013). The diversity of this genetic structure explains the difference between the genetic environments of these two *bla*_KPC−2_-carrying plasmids and why they appeared to form through different mechanisms. We therefore reasonably speculate that the transposition event mediated by Tn*3*-based transposon from Enterobacterales results in integration of the *bla*_KPC−2_-containing fragment into the backbones of inherent plasmids of *Aeromonas* spp., thereby generating the *bla*_KPC−2_-carrying plasmids like pAh2111-2-KPC. The establishment of another plasmid in our study was presumably due to horizontal transfer facilitated by insertion sequences flanking the *bla*_KPC−2_ gene without the Tn*3*-based transposons; alternatively, it is possible that genes encoding Tn*3*-transposase and its resolvase were lost upon transposition during the process of dissemination of this plasmid. Moreover, the genus *Aeromonas* is naturally transformable under optimal conditions, and most *Aeromonas* spp. prefer to acquire DNA from close relatives (Huddleston et al., 2013); therefore, the horizontal transferability between the *Aeromonas* spp. for these *bla*_KPC−2_-bearing plasmids is a concern if we need to prevent and control the dissemination of resistance elements in this genus.

## Conclusion

This study reported the identification of the *bla*_KPC−2_ gene located in a conserved genetic structure in two closed-related clinical isolates of *A. hydrophila* which shared a similar virulence gene profile. Such structure was most likely derived from Enterobacterales. Both *Aeromonas* isolates displayed high-level resistance to carbapenems encoded by both the chromosomal MBL gene *imiH* and two *bla*_KPC−2_ genes located in two distinct plasmids, one of which can undergo horizontal transfer between *Aeromonas* spp. To the best of our knowledge, this is the first report of clonal spread of KPC-producing *A. hydrophila* which carries two *bla*_KPC−2_-bearing plasmids in clinical settings. Hence, nosocomial surveillance for *bla*_KPC−2_ reservoirs should not be limited to Enterobacterales, but also other Gram-negative bacteria with standard workup established under the conception of “One Health.”

## Data Availability Statement

The datasets presented in this study can be found in online repositories. The names of the repository/repositories and accession number(s) can be found in the article/Supplementary Material.

## Author Contributions

JC and RZ conceived and designed the study. JC collected the *A. hydrophila* isolates. ZX and WS performed the experiments, analyzed the data, and drafted the manuscript. All authors revised the manuscript and approved the final version.

## Funding

This study was supported by the Zhejiang Provincial Natural Science Foundation of China (LY22H200001).

## Conflict of Interest

The authors declare that the research was conducted in the absence of any commercial or financial relationships that could be construed as a potential conflict of interest.

## Publisher's Note

All claims expressed in this article are solely those of the authors and do not necessarily represent those of their affiliated organizations, or those of the publisher, the editors and the reviewers. Any product that may be evaluated in this article, or claim that may be made by its manufacturer, is not guaranteed or endorsed by the publisher.

## Supplementary Material

Supplementary Table S1 Virulence factors detectable in the two KPC-2-producing *A. hydrophila* isolates.

## References

[B1] Alikhan N. F. Petty N. K. Ben Zakour N. L. Beatson S. A. (2011). BLAST Ring Image Generator (BRIG): simple prokaryote genome comparisons. BMC Genomics 12, 402. 10.1186/1471-2164-12-402 21824423 PMC3163573

[B2] Bankevich A. Nurk S. Antipov D. Gurevich A. A. Dvorkin M. Kulikov A. S.. (2012). SPAdes: a new genome assembly algorithm and its applications to single-cell sequencing. J. Comput. Biol. 19, 455–477. 10.1089/cmb.2012.0021 22506599 PMC3342519

[B3] Bush K. Bradford P. A. (2020). Epidemiology of β-lactamase-producing pathogens. Clin. Microbiol. Rev. 33, e00047-19. 10.1128/CMR.00047-19 32102899 PMC7048014

[B4] Cai J. C. Zhou H. W. Zhang R. Chen G. X. (2008). Emergence of *Serratia marcescens, Klebsiella pneumoniae*, and *Escherichia coli* isolates possessing the plasmid-mediated carbapenem-hydrolyzing β-lactamase KPC-2 in intensive care units of a Chinese hospital. Antimicrob. Agents Chemother. 52, 2014–2018. 10.1128/AAC.01539-07 18332176 PMC2415814

[B5] Carattoli A. Zankari E. Garcia-Fernandez A. Voldby Larsen M. Lund O. Villa L.. (2014). In silico detection and typing of plasmids using PlasmidFinder and plasmid multilocus sequence typing. Antimicrob. Agents Chemother. 58, 3895–3903. 10.1128/AAC.02412-14 24777092 PMC4068535

[B6] Clinical and Laboratory Standards Institute (2015). Methods for Antimicrobial Dilution and Disk Susceptibility Testing of Infrequently Isolated or Fastidious Bacteria, Approved Standard, Third Edition M45. Wayne, PA: Clinical and Laboratory Standards Institute. 10.1086/510431 17173232

[B7] Clinical and Laboratory Standards Institute (2021). Performance Standards for Antimicrobial Susceptibility Testing, 31th edition. CLSI Supplement M100. Wayne, PA: Clinical and Laboratory Standards Institute.

[B8] Giacobbe D. R. Del Bono V. Trecarichi E. M. De Rosa F. G. Giannella M. Bassetti M.. (2015). Risk factors for bloodstream infections due to colistin-resistant KPC-producing *Klebsiella pneumoniae*: results from a multicenter case-control-control study. Clin. Microbiol. Infect. 21, 1106 e1101–1108. 10.1016/j.cmi.2015.08.001 26278669

[B9] Hu X. Yu X. Shang Y. Xu H. Guo L. Liang Y.. (2019). Emergence and characterization of a novel IncP-6 plasmid harboring *bla*_KPC−2_ and *qnrS2* genes in *Aeromonas taiwanensis* isolates. Front. Microbiol. 10, 2132. 10.3389/fmicb.2019.02132 31572337 PMC6751286

[B10] Huddleston J. R. Brokaw J. M. Zak J. C. Jeter R. M. (2013). Natural transformation as a mechanism of horizontal gene transfer among environmental *Aeromonas* species. Syst. Appl. Microbiol. 36, 224–234. 10.1016/j.syapm.2013.01.004 23541366

[B11] Hughes H. Y. Conlan S. P. Lau A. F. Dekker J. P. Michelin A. V. Youn J. H.. (2016). Detection and whole-genome sequencing of carbapenemase-producing *Aeromonas hydrophila* isolates from routine perirectal surveillance culture. J. Clin. Microbiol. 54, 1167–1170. 10.1128/JCM.03229-15 26888898 PMC4809936

[B12] Janda J. M. Abbott S. L. (2010). The genus *Aeromonas*: taxonomy, pathogenicity, and infection. Clin. Microbiol. Rev. 23, 35–73. 10.1128/CMR.00039-09 20065325 PMC2806660

[B13] Mathys D. A. Mollenkopf D. F. Feicht S. M. Adams R. J. Albers A. L. Stuever D. M.. (2019). Carbapenemase-producing Enterobacteriaceae and *Aeromonas* spp. present in wastewater treatment plant effluent and nearby surface waters in the US. PLoS ONE 14, e0218650. 10.1371/journal.pone.0218650 31242271 PMC6594618

[B14] Montezzi L. F. Campana E. H. Correa L. L. Justo L. H. Paschoal R. P. da Silva I. L.. (2015). Occurrence of carbapenemase-producing bacteria in coastal recreational waters. Int. J. Antimicrob. Agents 45, 174–177. 10.1016/j.ijantimicag.2014.10.016 25499185

[B15] Munoz-Price L. S. Poirel L. Bonomo R. A. Schwaber M. J. Daikos G. L. Cormican M.. (2013). Clinical epidemiology of the global expansion of *Klebsiella pneumoniae* carbapenemases. Lancet Infect. Dis. 13, 785–796. 10.1016/S1473-3099(13)70190-7 23969216 PMC4673667

[B16] Nolla-Salas J. Codina-Calero J. Valles-Angulo S. Sitges-Serra A. Zapatero-Ferrandiz A. Climent M. C.. (2017). Clinical significance and outcome of *Aeromonas* spp. infections among 204 adult patients. Eur. J. Clin. Microbiol. Infect. Dis. 36, 1393–1403. 10.1007/s10096-017-2945-4 28258303 PMC7102105

[B17] Overbeek R. Olson R. Pusch G. D. Olsen G. J. Davis J. J. Disz T.. (2014). The SEED and the Rapid Annotation of microbial genomes using Subsystems Technology (RAST). Nucleic Acids Res. 42, D206–D214. 10.1093/nar/gkt1226 24293654 PMC3965101

[B18] Picao R. C. Cardoso J. P. Campana E. H. Nicoletti A. G. Petrolini F. V. Assis D. M.. (2013). The route of antimicrobial resistance from the hospital effluent to the environment: focus on the occurrence of KPC-producing *Aeromonas* spp. and *Enterobacteriaceae* in sewage. Diagn. Microbiol. Infect. Dis. 76, 80–85. 10.1016/j.diagmicrobio.2013.02.001 23478032

[B19] Sekizuka T. Inamine Y. Segawa T. Hashino M. Yatsu K. Kuroda M. (2019). Potential KPC-2 carbapenemase reservoir of environmental *Aeromonas hydrophila* and *Aeromonas caviae* isolates from the effluent of an urban wastewater treatment plant in Japan. Environ. Microbiol. Rep. 11, 589–597. 10.1111/1758-2229.12772 31106978 PMC6851574

[B20] Shen P. Wei Z. Jiang Y. Du X. Ji S. Yu Y.. (2009). Novel genetic environment of the carbapenem-hydrolyzing β-lactamase KPC-2 among Enterobacteriaceae in China. Antimicrob. Agents Chemother. 53, 4333–4338. 10.1128/AAC.00260-09 19620332 PMC2764158

[B21] Tang L. Huang J. She J. Zhao K. Zhou Y. (2020). Co-occurrence of the *bla*_KPC−2_ and *mcr-3.3* gene in *Aeromonas caviae* SCAc2001 isolated from patients with diarrheal disease. Infect. Drug Resist. 13, 1527–1536. 10.2147/IDR.S245553 32547122 PMC7259443

[B22] van Duin D. Doi Y. (2017). The global epidemiology of carbapenemase-producing Enterobacteriaceae. Virulence 8, 460–469. 10.1080/21505594.2016.1222343 27593176 PMC5477705

[B23] Wei Z. Q. Du X. X. Yu Y. S. Shen P. Chen Y. G. Li L. J. (2007). Plasmid-mediated KPC-2 in a *Klebsiella pneumoniae* isolate from China. Antimicrob. Agents Chemother. 51, 763–765. 10.1128/AAC.01053-06 17145797 PMC1797727

[B24] Wick R. R. Judd L. M. Gorrie C. L. Holt K. E. (2017). Unicycler: Resolving bacterial genome assemblies from short and long sequencing reads. PLoS Comput. Biol. 13, e1005595. 10.1371/journal.pcbi.1005595 28594827 PMC5481147

[B25] Xu H. Wang X. Yu X. Zhang J. Guo L. Huang C.. (2018). First detection and genomics analysis of KPC-2-producing *Citrobacter* isolates from river sediments. Environ. Pollut. 235, 931–937. 10.1016/j.envpol.2017.12.084 29358148

[B26] Yang J. Ye L. Guo L. Zhao Q. Chen R. Luo Y.. (2013). A nosocomial outbreak of KPC-2-producing *Klebsiella pneumoniae* in a Chinese hospital: dissemination of ST11 and emergence of ST37, ST392 and ST395. Clin. Microbiol. Infect. 19, E509–515. 10.1111/1469-0691.12275 23841705

[B27] Yigit H. Queenan A. M. Anderson G. J. Domenech-Sanchez A. Biddle J. W. Steward C. D.. (2001). Novel carbapenem-hydrolyzing β-lactamase, KPC-1, from a carbapenem-resistant strain of *Klebsiella pneumoniae*. Antimicrob. Agents Chemother. 45, 1151–1161. 10.1128/AAC.45.4.1151-1161.2001 11257029 PMC90438

[B28] Zankari E. Hasman H. Cosentino S. Vestergaard M. Rasmussen S. Lund O.. (2012). Identification of acquired antimicrobial resistance genes. J. Antimicrob. Chemother. 67, 2640–2644. 10.1093/jac/dks261 22782487 PMC3468078

